# Bar Attendance and Alcohol Use Before and After COVID-19 Related Restrictions Among HIV-infected Adults in South-Western Uganda

**DOI:** 10.1007/s10461-022-03934-9

**Published:** 2022-11-28

**Authors:** Caroline Asiimwe, Robin Fatch, Debbie M. Cheng, Nneka I. Emenyonu, Christine Ngabirano, Winnie R. Muyindike, Judith A. Hahn

**Affiliations:** 1 Global Health Collaborative, Mbarara University of Science and Technology, Mbarara, Uganda; 2 Department of Medicine, Department of Epidemiology, University of California, San Francisco, San Francisco, CA USA; 3 Boston University School of Public Health, Boston, MA USA; 4 Department of Internal Medicine, Mbarara Regional Referral Hospital, Mbarara, Uganda; 5 UCSF Mission Hall, 550 16th Street, Room 3550, UCSF Box 1224, 94158 San Francisco, CA USA

**Keywords:** Unhealthy alcohol use, COVID-19 restrictions, bar attendance, phosphatidylethanol, PLWH

## Abstract

Alcohol use is especially problematic for people living with HIV (PLWH) and was likely to be impacted by the coronavirus disease (COVID-19) pandemic and its restrictions. In a study of PLWH with latent tuberculosis infection, we measured unhealthy alcohol use with the Alcohol Use Disorders Identification Test (AUDIT-C), phosphatidylethanol (PEth) and bar attendance. We analyzed data collected before and after COVID-19 restrictions, and used Generalized Estimating Equations (GEE) logistic regression models to evaluate changes in unhealthy alcohol use. While bar attendance declined from 57.0% before to 38.3% after the restrictions started, multivariable analysis controlling for bar use showed a significant increase in unhealthy alcohol use; the adjusted odds ratio for unhealthy drinking before versus after the restrictions started was 1.37 (95% CI: 0.89–2.12) which increased to 1.64 (95% CI: 1.08–2.50) when bar attendance was added to the model. Decline in bar attendance did not decrease unhealthy alcohol use.

## Introduction

Unhealthy alcohol use is a major risk factor for HIV infection, onward transmission [1–4], and poor treatment outcomes [5–11]. Unhealthy alcohol use is defined as drinking levels above safe health limits, e.g. defined by National Institute on Alcohol Abuse and Alcoholism as drinking no more than one drink a day for women, and no more than two drinks a day for men [12]. Unhealthy alcohol use is common in Sub-Saharan Africa (SSA), where the majority of persons living with HIV (PLWH) reside. This is especially true in Uganda, where the HIV prevalence is 6.2% [13], 60% of Ugandans drink alcohol and 56.3% of those engage in heavy episodic drinking [14]. Therefore, reduction of unhealthy alcohol use among PLWH is crucial.

There has been a rapid emergence of coronavirus disease (COVID-19) throughout the world, and in March 2020 the Ugandan government in collaboration with the Ministry of Health instituted restrictions in order to contain the corona virus spread [15]. Those restrictions included bar closures, stay-at-home instructions for non-essential workers, a ban on public transport that later operated at 50% capacity, and a ban on road travel between the hours of 19:00 and 06:00 [15]. Restrictions on travel were lifted on 04/06/2020 and re-imposed again from 10th June 2021 to 31st July 2021; bars were officially closed from 18 March 2020 until 24 January 2022.

Several studies conducted during COVID-19 restrictions and stay-at-home orders/quarantine have shown an increase in alcohol use [16–18]. However, other studies reported a decline in alcohol use [19, 20]. The COVID-19 pandemic has been associated with anxiety, stress, frustration, social isolation and depression, which are likely to impact alcohol use as a means of self-medication [17, 21, 22] and as a coping mechanism [23]. PLWH are of concern because high rates of alcohol consumption and alcohol related problems have been reported among this group [24–26]. The COVID-19 pandemic has had deleterious effects on health facility access to ART [27], possibly affecting antiretroviral (ART) medication adherence. PLWH who engage in alcohol use are at high risk of ART non-adherence, thus trends in alcohol use due to COVID-19 restrictions are important to study.

Although several studies have reported on alcohol use during the COVID-19 restrictions [17–20], information on attending drinking establishments such as bars is limited, especially among PLWH. Studies conducted prior to COVID-19 have shown a significant and positive relationship between bar attendance and the amount of alcohol consumed [28, 29]. Despite the Ugandan restrictions on bar openings during COVID-19, it is not known whether some bars served customers despite the restrictions. In addition, alcohol was also available in some shops and supermarkets, which were considered providers of important services to the communities. Thus, the extent to which bar attendance and alcohol consumption changed during the different periods before and after COVID-19 restrictions is not known.

The study aimed to (1) describe bar attendance and unhealthy alcohol use before COVID-19 restrictions among PLWH (Aim 1), (2) describe bar attendance and unhealthy alcohol use before and after COVID-19 restrictions started (before and after March 2020) (Aim 2), and (3) examine the impact of COVID-19 restrictions on unhealthy alcohol use among PLWH (Aim 3).

## Methods

### Study Design and Setting

We conducted the analysis using data from the Alcohol Drinkers Exposure to Preventive Therapy for Tuberculosis (ADEPTT, NCT03302299) Study, a prospective cohort study which aimed at examining the safety and tolerability of isoniazid (INH) preventive therapy (IPT) in persons with HIV and latent tuberculosis (TB) co-infection who drink alcohol and those that do not drink alcohol. The study was done at the Immune Suppression Syndrome (ISS) HIV clinic within Mbarara Regional Referral Hospital in Southwestern Uganda. This study is part of the Uganda-Russia-Boston Alcohol Network for Alcohol Research Collaboration on HIV/AIDS (URBAN ARCH) Consortium.

### Study Participants and Procedures

Patients were eligible for the ADEPTT study if they reported either current alcohol use (within the prior three months) or abstaining from alcohol (no alcohol use in the prior year). Participants were purposively enrolled in a ratio of two persons reporting current alcohol use to one person reporting abstaining. The study enrolled adults (≥ 18 years old) living with HIV, who were fluent in either Runyankole (the local language) or English, had been on antiretroviral therapy (ART) for at least 6 months, lived within 60 km of the study site and had no plans to move out of the catchment area, and who had no history of active TB or taking TB preventive medications. Exclusion criteria included having taken nevirapine (NVP) in the prior 2 weeks (due to its known toxicity when combined with INH); taking anti-convulsant medications; having alanine transaminase (ALT) or aspartate transaminase (AST) levels > 2x the upper limit of normal (baseline study test); active TB (for those reporting TB symptoms); and being pregnant. Eligible participants were screened for latent TB using a tuberculin skin test (TST) and invited for the prospective study if the TST was positive, defined as having an induration ≥ 5 mm 48–72 hours after injection with purified protein derivative (PPD). We conducted interviews at baseline, 3 months, 6 months and bi-yearly after that, for up to 42 months of follow-up. For this analysis, we examined only those who reported any current alcohol use at baseline and who had at least one visit prior to the restrictions and one visit following the restrictions. Face-to-face interviews were conducted from May 2017 to March 2021. Due to COVID-19 restrictions, interviews were halted from March 2020 to June 2020.

### Ethical Considerations

All study procedures were approved by the Institutional Review Boards at University of California San-Francisco (UCSF), Mbarara University of Science and Technology (MUST) and Uganda National Council of Science and Technology (UNCST). The participants gave written informed consent to participate in the study.

### Study Survey

We conducted interviewer administered structured surveys at each study visit using the Computer Assisted Survey Information Collection (CASIC) system assessment. The participants were interviewed in English or Runyakole depending on the participant’s preference. The survey included demographic variables, self-reported alcohol consumption, measured by the 3-question Alcohol Use Disorders Identification Test-Consumption (AUDIT-C) [31] (modified to cover the prior 3 months), lifetime drinking, amount and type of alcohol used/consumed, drinking locations, and drinking companions.

### Specimen Collection

Blood was collected at baseline, 3 months, 6 months and bi-yearly via a venous blood draw. Dried blood spot (DBS) cards were prepared to test for phosphatidylethanol (PEth), a biomarker of alcohol consumption in the prior 2–4 weeks [24]. Phosphatidylethanols are a group of abnormal phospholipids that are formed only in the presence of ethanol (alcohol), and PEth has been shown in several studies to be correlated with the amount of alcohol taken, with Spearman correlations above 0.50 [30]. DBS cards were tested for PEth (16:0/18:1 homologue, limit of quantification 8 ng/mL) at the United States Drug Testing Laboratories Inc [31].

### Key Variables

Bar attendance: At each visit, we measured bar attendance by the question on the survey which read, “Where did you usually drink alcohol in the last 3 months?” Participants were asked to choose the location(s) in which they usually drank alcohol; the options were bars, parties or celebrations, restaurants, homes, and/or somewhere else. Participants were allowed to choose more than one drinking location. We dichotomized responses to this question based on whether the participant reported drinking at bars.

Unhealthy alcohol use: Because we previously have found alcohol use to be under-reported in this population, we used self-report combined with PEth for our measure of unhealthy alcohol use. This was defined as a positive score on the AUDIT-C of ≥ 3 or ≥ 4 for females and males respectively, modified to cover the prior 3 months, or a PEth value of ≥ 50 ng/mL. The PEth ≥ 50 ng/ml cutoff was based on previous literature [24, 32] that this would yield good sensitivity and specificity for unhealthy alcohol use. The combination of self-report with PEth was designed to take advantage of the high specificity of each measure and has been used in previous studies to detect unhealthy alcohol use [33].

COVID-19 restrictions: We indicated COVID-19 restrictions using the date of the study visit, either prior to the restrictions (before 18th March 2020) or after the restrictions (after 26th May 2020) were gradually lifted.

Covariates: Covariates of interest included participants’ age, gender, marital status, education, current (prior 3 months) tobacco use at baseline, current (prior 3 months) other substance use at baseline, and time in study (months). These variables were selected based on clinical knowledge and literature, as they have previously shown an association with unhealthy alcohol use [26, 34].

### Statistical Analysis

Sample characteristics were described using proportions for categorical variables, means, standard deviations, medians and inter-quartile range (IQR) for continuous variables. We used baseline data (i.e. data from participants’ first ADEPTT study interview) for participant characteristics (age, gender, marital status, level of education, tobacco use and other substances) and data collected at each study visit for alcohol use and bar attendance.

We examined variables associated with unhealthy alcohol use and with identifying bars as a usual drinking location, and we graphed bar attendance and unhealthy alcohol use before and after COVID-19 restrictions by study time period, grouping calendar time by 6-month. The early time periods in the graphs reflect the beginning of the study, when recruitment was ongoing; as such the sample size was smaller during these periods, as is noted in the figures.

We conducted Generalized Estimating Equations (GEE) logistic regression modeling with robust standard errors to examine the association between presence of COVID-19 restrictions and unhealthy alcohol use, using all study visits for each participant. The primary multivariable model of interest was adjusted for age, gender, marital status, education, current tobacco use at baseline, and time in study (months). “Other substance use” at baseline was uncommon and was thus excluded from the multivariable models due to concern about small cell sizes. In exploratory analyses, we also: (1) tested the interaction between time in study and the institution of COVID-19 restrictions, to account for a potential study effect over time, and (2) adjusted the primary model for bar attendance to explore its role (i.e. confounder or mediator) in the relationship between presence of COVID-19 restrictions and unhealthy alcohol use.

## Results

### Baseline Characteristics

We recruited 200 persons in the ADEPTT study who reported current alcohol use, and 193 had at least one visit before COVID-19 restrictions and at least one visit after the institution of restrictions. Out of 193 participants, 118 (61.1%) were male, 136 (70.5%) were married and 136 (70.5%) had not completed primary education. In their last visit prior to the start of restrictions, 127 (65.8%) participants engaged in unhealthy alcohol use and 109 (56.5%) reported bar attendance as their main drinking location. Thirty persons (15.5%) reported tobacco use and ten (5.2%) reported using other substances in the prior 3 months at baseline (Table I).

**Table I:** Study participant characteristics, overall and by unhealthy alcohol use and bar as usual site of alcohol consumption at the last study visit prior to implementation of COVID-19 restrictions. The sample was restricted to those who reported any prior 3 months alcohol use at baseline and who had at least one visit before and after COVID-19 restrictions were implemented (n = 193)

	**Overall** **N (%)**	**Unhealthy alcohol use (AUDIT-C positive and/or PEth ≥ 50 ng/ml)**, **last visit prior to the restrictions**		**Bar as usual site of alcohol consumption, last visit prior to the restrictions**	
		**No**	**Yes**	**No**	**Yes**
Age (median [IQR])	39 [32–45]	39 [32–44]	39 [32–47]	40 [34–45]	38 [32–46]
Gender					
Female	75 (38.9)	41 (55.4)	33 (44.6)	47 (63.5)	27 (36.5)
Male	118 (61.1)	25 (21.0)	94 (79.0)	37 (31.1)	82 (68.9)
Married					
No	57 (29.5)	22 (38.6)	35 (61.4)	25 (43.9)	32 (56.1)
Yes	136 (70.5)	44 (32.4)	92 (67.7)	59 (43.4)	77 (56.6)
More than a primary education					
No	136 (70.5)	43 (31.6)	93 (68.4)	57 (41.9)	79 (58.1)
Yes	57 (29.5)	23 (40.4)	34 (59.7)	27 (47.4)	30 (52.6)
Tobacco use, prior 3 months, at baseline					
No	163 (84.5)	63 (38.7)	100 (61.4)	72 (44.2)	91 (55.8)
Yes	30 (15.5)	3 (10.0)	27 (90.0)	12 (40.0)	18 (60.0)
Other substance use, prior 3 months, at baseline					
No	183 (94.8)	63 (34.4)	120 (65.6)	81 (44.3)	102 (55.7)
Yes	10 (5.2)	3 (30.0)	7 (70.0)	3 (30.0)	7 (70.0)
Unhealthy alcohol use (AUDIT-C positive and/or PEth ≥ 50 ng/ml), last visit prior to the restrictions					
No	66 (34.2)	-	-	48 (72.7)	18 (27.3)
Yes	127 (65.8)	-	-	36 (28.4)	91 (71.7)
Where did you usually drink alcohol in the prior 3 months, last visit prior to the restrictions					
Bars					
No	84 (43.5)	48 (57.1)	36 (42.9)	-	-
Yes	109 (56.5)	18 (16.5)	91 (83.5)	-	-
Home					
No	146 (75.7)	52 (35.6)	94 (64.4)	51 (34.9)	95 (65.1)
Yes	47 (24.4)	14 (29.8)	33 (70.2)	33 (70.2)	14 (29.8)
Parties					
No	172 (89.1)	57 (33.1)	115 (66.9)	72 (41.9)	100 (58.1)
Yes	21 (10.9)	9 (42.9)	12 (57.1)	12 (57.1)	9 (42.9)

### Descriptive Analyses of Unhealthy Alcohol use (before-restrictions)

The median age of those who engaged in unhealthy alcohol use was similar to those who did not engage in unhealthy use (39 vs. 39, Table I). Unhealthy alcohol use was more common among the men compared to women; of the 118 men, 94 (79.0%) engaged in unhealthy alcohol use while 33 (44.6%) of the women engaged in unhealthy use. Unhealthy alcohol use was more common among those reporting bars as their main drinking location versus other locations (83.5% versus 42.9%).

### Descriptive analyses of bar attendance (before-restrictions)

The median age for participants who reported bars as their main site of alcohol use was similar to those who did not report bar attendance as their main drinking location (38 vs. 40). Reporting bars as the main site of drinking was more common among the men (68.9%) compared to the women (36.5%). We observed no differences in reporting bars as the main site of alcohol use by marital status or level of education (Table I).

### Unhealthy Alcohol use and bar Attendance Before and after-restrictions

**Fig. 1:**
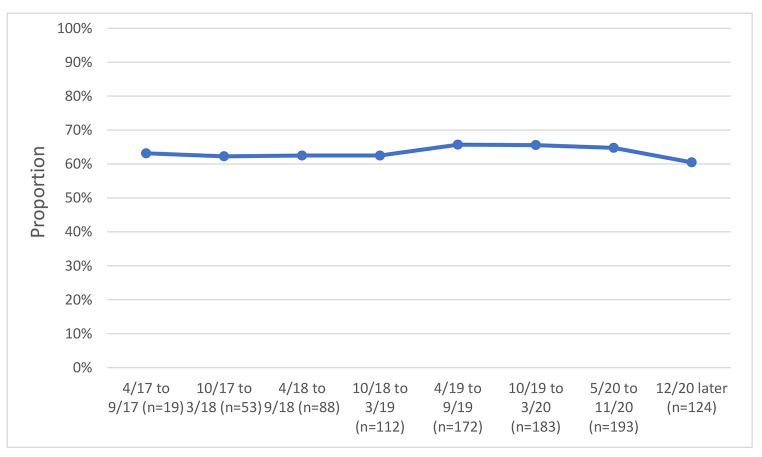
Unhealthy alcohol use by calendar time among 193 PLWH with at least one study visit before and after COVID-19 restrictions were implemented

**Fig. 2:**
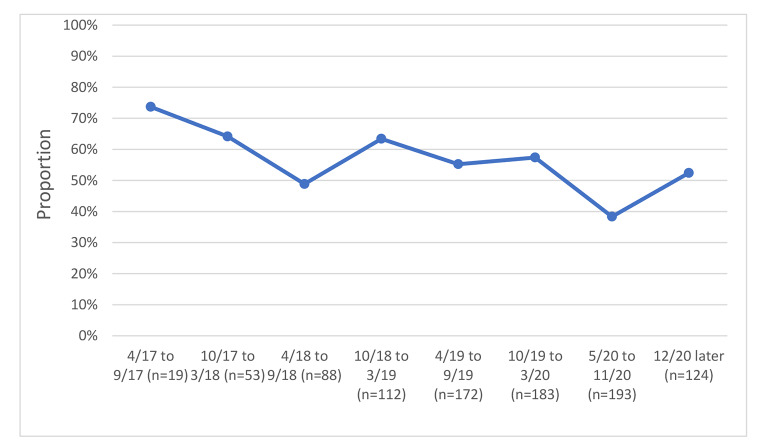
Bars as usual site of alcohol consumption by calendar time among 193 PLWH with at least one study visit before and after COVID-19 restrictions were implemented

**Fig. 3:**
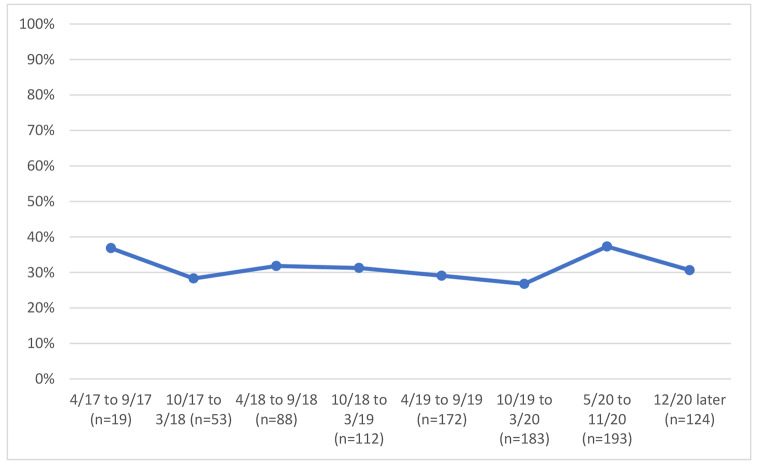
Home as usual site of alcohol consumption by calendar time among 193 PLWH with at least one study visit before and after COVID-19 restrictions were implemented

**Fig. 4:**
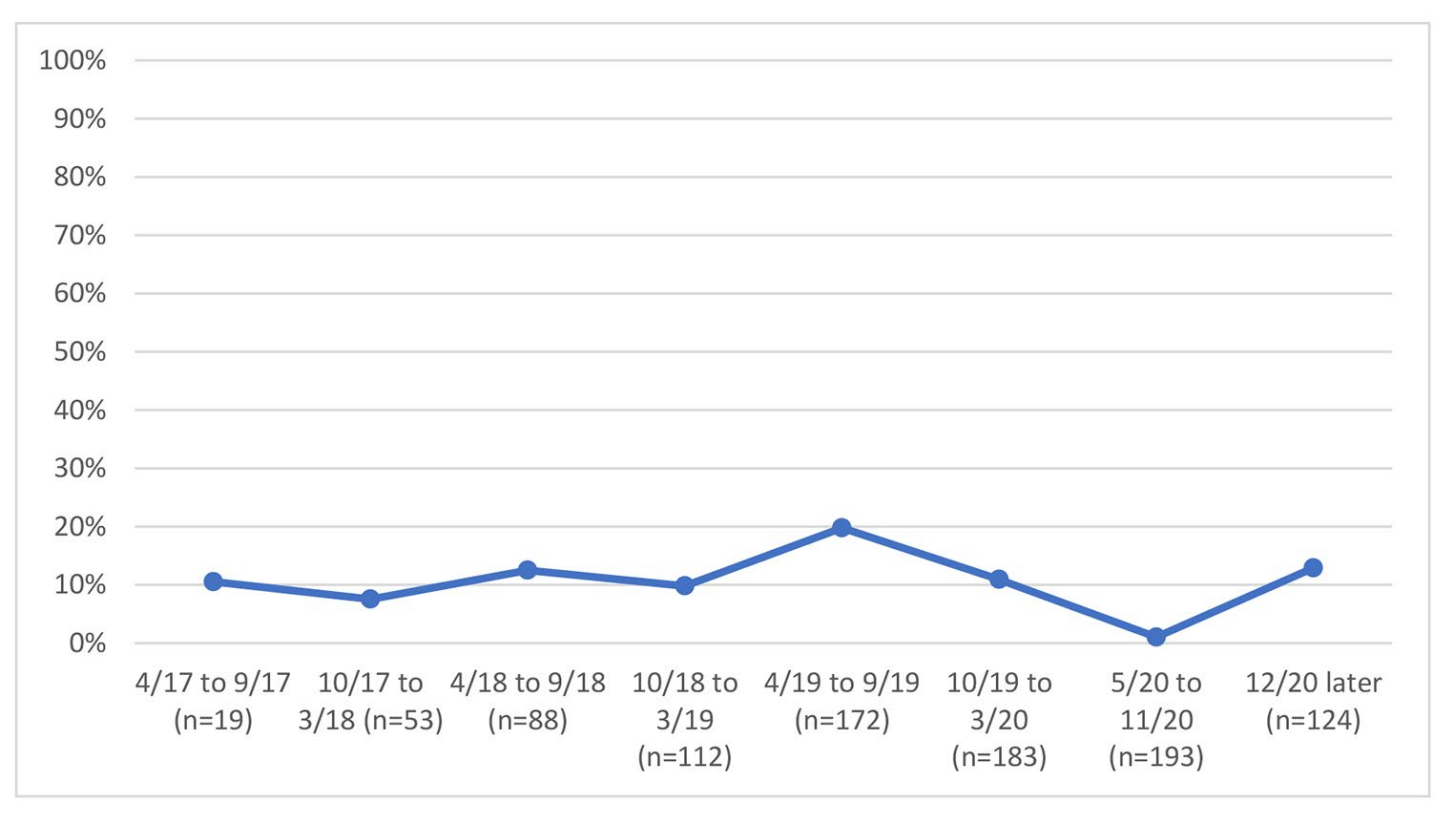
Parties as usual site of alcohol consumption by calendar time among 193 PLWH with at least one study visit before and after COVID-19 restrictions were implemented

We examined unhealthy alcohol use and bar attendance before and after the COVID-19 restriction period; in unadjusted analyses, we observed a 1% reduction in an unhealthy alcohol use by calendar time just before and just after COVID-19 restrictions (before and after March 2020) (Fig. 1), from 65.8 to 64.8%. Concurrently, we observed a decline in reporting bars as the primary drinking venue by calendar time before and after COVID-19 restrictions (before and after March 2020) (Fig. 2), from 57.0% at the last visit just before restrictions to 38.3% at the first visit just after restrictions. Changes in the prevalence of reporting venues other than bars occurred; homes were reported as the usual place of drinking by 26.4% before restrictions vs. 37.3% after restrictions (Fig. 3) and parties were reported as the usual place of drinking by 10.4% before restrictions and 1.0% after restrictions (Fig. 4).

### COVID-19 Restrictions and Unhealthy Alcohol use

#### Unadjusted Results

There was no evidence of an association between COVID-19 restrictions and unhealthy alcohol use (odds ratio [OR] 1.02, 95% confidence interval [CI] 0.84–1.24, z = 0.19, p = 0.85) in unadjusted analyses.

***Adjusted results***: In the primary analyses adjusting for potential confounders, the adjusted odds for unhealthy alcohol use after the restrictions was 1.37 times the odds before the restrictions (95% CI: 0.89–2.12, z = 1.42, p = 0.15) (Table II). Male gender and use of tobacco were associated with unhealthy alcohol use in both.

**Table II:** Unadjusted and adjusted odds ratios (OR) and 95% confidence intervals (CI) for unhealthy alcohol use in the prior 3 months (AUDIT-C positive and/or PEth ≥ 50 ng/ml). The sample was comprised of persons who reported any prior 3 months alcohol use at baseline and who had at least one study visit before and after COVID-19 restrictions were implemented (n = 193 participants and n = 1209 observations)

	**Unadjusted**		**Model 1**		Model 2*		
	**OR (95% CI)**	**z-statistic (p-value)**	**Adjusted OR (95% CI)**	**z-statistic** **(p-value)**	**Adjusted OR (95% CI)**	**z-statistic (p-value)**	
COVID-19 restrictions							
Prior to	1.00		1.00		1.00		
Post	1.02 (0.84, 1.24)	0.19 (0.85)	1.37 (0.89, 2.12)	1.42 (0.15)	1.64 (1.08, 2.50)	2.33 (0.02)	
Age (per 1 year)	1.00 (0.98, 1.03)	0.03 (0.98)	0.98 (0.95, 1.01)	-1.44 (0.15)	0.99 (0.96, 1.02)	-0.82 (0.41)	
Gender							
Female	1.00		1.00		1.00		
Male	3.71 (2.24, 6.12)	5.11 (< 0.01)	4.03 (2.18, 7.45)	4.46 (< 0.01)	2.26 (1.19, 4.29)	2.48 (0.01)	
Married							
No	1.00		1.00		1.00		
Yes	1.15 (0.68, 1.96)	0.52 (0.60)	0.89 (0.50, 1.60)	-0.39 (0.70)	1.10 (0.62, 1.95)	0.34 (0.73)	
More than a primary education							
No	1.00		1.00		1.00		
Yes	0.70 (0.41, 1.22)	-1.25 (0.21)	0.65 (0.37, 1.16)	-1.45 (0.15)	0.73 (0.40, 1.30)	-1.07 (0.28)	
Tobacco use, at baseline							
No	1.00		1.00		1.00		
Yes	4.00 (1.99, 8.04)	3.89 (< 0.01)	2.57 (1.23, 5.39)	2.50 (0.01)	3.14 (1.40, 7.01)	2.79 (< 0.01)	
Bar as main site for alcohol consumption, prior 3 months							
No	1.00		-		1.00		
Yes	4.82 (3.33, 6.98)	8.32 (< 0.01)	-		3.88 (2.64, 5.69)	6.93 (< 0.01)	
Time in study (per 1 month)	1.00 (0.99, 1.01)	-0.52 (0.61)	0.98 (0.96, 1.00)	-1.55 (0.12)	0.99 (0.97, 1.01)	-1.34 (0.18)	

* Model 2 is adjusted for the same variables as Model 1, but is additionally adjusted for bar as main site of alcohol consumption in the prior 3 months.

The interaction between time and COVID-19 restrictions was not significant and was therefore excluded from the final models. In exploratory analyses further adjusting for bar attendance, the adjusted OR (aOR) for COVID-19 restriction was 1.64 (95% CI: 1.08–2.50, z = 2.33, p = 0.02).

## Discussion

We examined the impact of COVID-19 restrictions on unhealthy alcohol use among PLWH in Uganda. Overall, we found minimal change in the proportion that reported unhealthy alcohol use from before and after the 2020 restrictions in unadjusted analyses, but found a significant increase in analyses adjusted for bar attendance. Two-thirds of the study participants reported unhealthy alcohol use before (65%) and after (66%) the 2020 COVID-19 related restrictions. The findings suggest that the institution of bar-related restrictions did not reduce unhealthy alcohol use, but when we additionally controlled for bar attendance, the association between COVID-19 restrictions and unhealthy alcohol use increased. This unexpected finding could have been a result of alcohol accessibility from shops and supermarkets which remained open during the COVID-19 period. This study finding is comparable to other studies that found an increase in alcohol use [17, 18, 35], although other studies showed a decline in unhealthy alcohol use in COVID-19 times [19, 20]. A meta-analysis study also revealed no changes in drinking during the pandemic [36]. This shows a likely complex relationship between the restrictions and drinking with some factors associated with increased and others with decreased drinking which explains the lack of consistency in results worldwide.

Bars were frequently the usual source of drinking for those with unhealthy alcohol use. Their use declined from before to after COVID-19 restrictions but this did not seem to be associated with decreases in unhealthy alcohol use. Bars were the most common locations for drinking prior to the restrictions, especially by men. The odds of drinking in bars for men was twice that of women. This is consistent with other studies that report that men drink in public places to interact and socialize with other men [37]. Although bars were officially closed, other venues like shops and supermarkets that were allowed to operate during the restrictions could have increased the availability of alcohol, and it is likely, given that 38.5% still reported bars as their main drinking site, that some bars found a way to operate surreptitiously. Despite the mandated bar closure, alcohol was still available and accessible to many during COVID-19 restrictions. We note that all persons included in this study were tuberculin skin test (TST) positive and were PLWH, thus they were at high risk of active TB. The high level of bar attendance, both before and during the restrictions, is of concern because bars are a likely site of TB disease transmission [38, 39].

Homes were the second most common location in which people consumed alcohol; 26.6% before COVID-19 restrictions vs. 37.5% after the restrictions. Unlike at parties where alcohol consumption reduced, likely due to restrictions on public gatherings, there was increased consumption in homes during the COVID-19 restrictions period. This could have been attributed to most people being under the stay-home restriction, necessitating them to work from home thus creating more convenience and time to consume alcohol. Our results suggest that the closure of bars by the government of Uganda did not necessarily stop people from attending bars and or finding other venues for alcohol consumption. While bars were officially closed, there was no reduction in unhealthy drinking. That suggests that attempts to reduce alcohol use with restrictive structural interventions without addressing individual motivations to drink are unlikely to be successful in reducing alcohol use.

This study had limitations. All people included in this study were PLWH with latent TB infection that consume alcohol. Because of these restrictive eligibility criteria, the findings may not be generalizable to the general population of PLWH. Bar attendance was collected by self-report; not all bar attendance may have been reported. Due to recall and misclassification biases, participants may have substantially mis-reported, likely in the direction of under-report, their bar attendance in this study, especially after the restrictions began. We may have also missed occasional drinking at bars. The bar attendance variable measured bars as a usual place of alcohol consumption rather than any bar attendance.

Finally, this exploratory, hypothesis-generating study was likely underpowered to detect significant associations. In post hoc power calculations, we estimated that 18% of participants changed unhealthy drinking status before vs. after COVID-19 restrictions based on our observed data. Under this assumption, the study has approximately 80% power to detect an odds ratio as small as 2.9, suggesting the study was underpowered to detect effects of the observed magnitude.

Regardless of the above limitations, there are several strengths of note. First, the study population included the same people before and after the COVID-19 restrictions. Other studies that were conducted had their baseline data collected during the period of COVID-19 restrictions, thus, they were unable to ascertain the trend of unhealthy alcohol use. In addition, we used PEth combined with self-report to minimize issues of under reporting alcohol use [40–42]. Above all, this is the first study in Uganda to study bar attendance and unhealthy alcohol use before and after COVID-19 restrictions.

## Conclusion

In this study, we did not observe a reduction in unhealthy alcohol use after the implementation of COVID-19 restrictions. Notably, despite the decline of bar attendance during the COVID-19 restrictions, adjustment for this factor suggested a positive association between COVID-19 restrictions and unhealthy alcohol use, and a concurrent increase in drinking from other places like homes. While bars were officially closed, there was no reduction in unhealthy drinking. That suggests that attempts to reduce alcohol use with restrictive structural interventions without addressing individual motivations to drink are unlikely to be successful in reducing alcohol use. Individual interventions to improve unhealthy alcohol use and limit bar attendance especially during pandemics are needed to improve health outcomes. Intervening on alcohol use itself may be needed, for example, interventions that help to motivate behavior change.

## Data Availability

The data analyzed for this manuscript will be made available via upload to the Dryad Digital Repository upon acceptance for publication.

## References

[1] Shuper PA, Neuman M, Kanteres F, Baliunas D, Joharchi N, Rehm J (2010). Causal considerations on alcohol and HIV/AIDS - A systematic review. Alcohol Alcohol.

[2] Hahn JA, Woolf-King SE, Muyindike W (2011). Adding fuel to the fire: Alcohol’s effect on the HIV epidemic in sub-saharan africa. Curr HIV/AIDS Rep.

[3] Rehm J, Gmel GE, Gmel G (2017). The relationship between different dimensions of alcohol use and the burden of disease—an update. Addiction.

[4] Woolf-king SE, Fatch R, Cheng DM, Ngabirano C, Kekibiina A, Hahn JA (2019). Adults: findings from an event-level study. Arch Sex Behav.

[5] Baliunas D, Rehm J, Irving H, Shuper P (2010). Alcohol consumption and risk of incident human immunodeficiency virus infection: a meta-analysis. Int J Public Health.

[6] Kim JY, Yang Y, Kim HK (2018). The impact of Alcohol Use on Antiretroviral Therapy Adherence in Koreans living with HIV. Asian Nurs Res (Korean Soc Nurs Sci).

[7] Neuman MG, Schneider M, Nanau RM, Parry C. Alcohol consumption, progression of disease and other comorbidities, and responses to antiretroviral medication in people living with HIV. AIDS Research and Treatment. 2012. Available from: https://www.hindawi.com/journals/art/2012/751827/. 10.1155/2012/751827 PMC3310201 22496971

[8] Pokhrel KN, Pokhrel GK, Neupane SR, Sharma VD. Harmful alcohol drinking among HIV-positive people in Nepal: an overlooked threat to anti-retroviral therapy adherence and health-related quality of life. Global Health Action. Taylor & Francis; 2018. Available from: 10.1080/16549716.2018.1441783. 10.1080/16549716.2018.1441783 PMC5844022 29495948

[9] Lesko CR, Keil AP, Fojo AT, Chander G, Lau B, Moore RD (2019). Recent substance Use and Probability of Unsuppressed HIV viral load among persons on antiretroviral therapy in Continuity Care. Am J Epidemiol.

[10] Williams CE, Hahn AJ, Saitz R, Bryant K, Lira CM, Samet HJ (2016). Alcohol Use and Human Immunodeficiency Virus (HIV) infection: current knowledge, implications, and future directions. Alcohol Clin Exp Res.

[11] Braithwaite RS, Bryant KJ (2010). Influence of alcohol consumption on adherence to and toxicity of antiretroviral therapy and survival. Alcohol Res Heal.

[12] National Institute on Alcohol Abuse and Alcoholism (NIAAA). Drinking Levels Defined. [cited 2022 Apr 25]. Available from: https://www.niaaa.nih.gov/alcohol-health/overview-alcohol-consumption/moderate-binge-drinking.

[13] Uganda AIDS, Commission (2017). Uganda Population based HIV Impact Assessment: UPHIA 2016–2017.

[14] WHO. Global Status Report on Alcohol and Health. Geneva; 2018.

[15] Ayebale. President Museveni’s full address on coronavirus. The Independent. 2020. Available from: https://www.independent.co.ug/president-musevenis-full-address-on-coronavirus/.

[16] Wu P, Liu X, Fang Y (2008). Alcohol abuse/dependence symptoms among hospital employees exposed to a SARS outbreak. Alcohol Alcohol.

[17] Pollard MS, Tucker JS, Green HD (2020). Changes in adult Alcohol Use and Consequences during the COVID-19 pandemic in the US. JAMA Netw open.

[18] Szajnoga D, Klimek-Tulwin M, Piekut A (2020). COVID-19 lockdown leads to changes in alcohol consumption patterns. Results from the polish national survey. J Addict Dis.

[19] Sallie SN, Ritou V, Bowden-Jones H, Voon V (2020). Assessing international alcohol consumption patterns during isolation from the COVID-19 pandemic using an online survey: highlighting negative emotionality mechanisms. BMJ Open.

[20] Chodkiewicz J, Talarowska M, Miniszewska J, Nawrocka N, Bilinski P (2020). Alcohol consumption reported during the COVID-19 pandemic: the initial stage. Int J Environ Res Public Health.

[21] Ahmed Z, Ahmed O, Aibao Z, Hanbin S, Siyu L, Ahmad A (2020). Epidemic of COVID-19 in China and associated psychological problems. Asian J Psychiatr.

[22] Brooks SK, Webster RK, Smith LE (2020). The psychological impact of quarantine and how to reduce it: rapid review of the evidence. Lancet.

[23] Esterwood E, Saeed SA, Past Epidemics N, Disasters (2020). COVID19, and Mental Health: learning from history as we deal with the Present and prepare for the future. Psychiatr Q.

[24] Hahn JA, Dobkin LM, Mayanja B (2012). Phosphatidylethanol (PEth) as a biomarker of alcohol consumption in HIV positives in sub-saharan Africa. Alcohol Clin Exp Res.

[25] Moss da Silva C, Mendoza-Sassi AR, Dias da Mota L, Nader MM, Barral de Martinez MA (2017). Alcohol use disorders among people living with HIV / AIDS in Southern Brazil: prevalence, risk factors and biological markers outcomes. BMC Infect Dis.

[26] Kabwama SN, Ndyanabangi S, Mutungi G, Wesonga R, Bahendeka SK, Guwatudde D (2016). Alcohol use among adults in Uganda: findings from the countrywide non-communicable diseases risk factor cross-sectional survey. Glob Health Action.

[27] Zakumumpa H, Tumwine C, Milliam K, Spicer N (2021). Dispensing antiretrovirals during Covid-19 lockdown: re-discovering community-based ART delivery models in Uganda. BMC Health Serv Res.

[28] Mills A, Caetano R, Vaeth ACP, Gonzalez MJR (2015). Disentangling contributions of Bar Attendance, drinking, and other factors to elevated acute alcohol problems on the U.S.- Mexico Border. Alcohol Clin Exp Res.

[29] Freisthler B, Alcohol, Use (2011). Drinking venue utilization, and child physical abuse: results from a pilot study. J Fam Violence.

[30] Ulwelling W, Smith K (2018). The PEth Blood Test in the security environment: what it is; why it is important; and interpretative guidelines. J Forensic Sci.

[31] Jones J, Jones M, Plate C, Lewis D (2011). The detection of 1-palmitoyl-2-oleoyl-sn-glycero-3-phosphoethanol in human dried blood spots. Anal Methods.

[32] Stewart HS, Law LT, Randall KP, Newman R (2010). Phosphatidylethanol and Alcohol Consumption in Reproductive Age Women. Alcohol Clin Exp Res.

[33] Hahn AJ, Emenyonu IN, Fatch R (2016). Declining and rebounding unhealthy alcohol consumption during the first year of HIV care in rural Uganda, using phosphatidylethanol to augment self-report. Addiction.

[34] Tumwesigye MZ, Kasirye R (2005). Gender and the major consequences of alcohol consumption in Uganda. Alcohol, gender and drinking problems: perspectives from Low and Middle Income Countries.

[35] Killgore WDS, Cloonan SA, Taylor EC, Lucas DA, Dailey NS (2021). Alcohol dependence during COVID-19 lockdowns. Psychiatry Res.

[36] Acuff SF, Strickland JC, Tucker JA, Murphy JG (2022). Changes in Alcohol Use during COVID-19 and Associations with Contextual and individual difference variables: a systematic review and Meta-analysis. Psychol Addict Behav.

[37] Wilsnack RW, Vogeltanz ND, Wilsnack SC (2000). Gender differences in alcohol consumption and adverse drinking consequences: cross-cultural patterns. Addiction.

[38] Kline SE, Hedemark LL, Davies SF (1995). Outbreak of tuberculosis among regular patrons of a Neighborhood Bar. N Engl J Med.

[39] Godoy P, Alsedà M, Falguera M (2017). A highly transmissible tuberculosis outbreak: the importance of bars. Epidemiol Infect.

[40] Muyindike WR, Lloyd-Travaglini C, Fatch R (2017). Phosphatidylethanol confirmed alcohol use among ART-naïve HIV-infected persons who denied consumption in rural Uganda. AIDS Care - Psychol Socio-Medical Asp AIDS/HIV.

[41] Bajunirwe F, Haberer JE, Boum Y (2014). Comparison of self-reported alcohol consumption to phosphatidylethanol measurement among HIV-infected patients initiating antiretroviral treatment in Southwestern Uganda. PLoS ONE.

[42] Papas KR, Gakinya NB, Mwaniki MM (2016). Associations between the phosphatidylethanol (PEth) alcohol biomarker and self-reported alcohol use in a sample of HIV-infected outpatient drinkers in western Kenya. Alcohol Clin Exp Res.

